# Systematic discovery of transcription factors that improve hPSC-derived cardiomyocyte maturation via temporal analysis of bioengineered cardiac tissues

**DOI:** 10.1063/5.0137458

**Published:** 2023-05-25

**Authors:** Aditya Kumar, Starry He, Prashant Mali

**Affiliations:** Department of Bioengineering, University of California, San Diego, California 92093, USA

## Abstract

Human pluripotent stem cell-derived cardiomyocytes (hPSC-CMs) have the potential to become powerful tools for disease modeling, drug testing, and transplantation; however, their immaturity limits their applications. Transcription factor (TF) overexpression can improve hPSC-CM maturity, but identifying these TFs has been elusive. Toward this, we establish here an experimental framework for systematic identification of maturation enhancing factors. Specifically, we performed temporal transcriptome RNAseq analyses of progressively matured hPSC-derived cardiomyocytes across 2D and 3D differentiation systems and further compared these bioengineered tissues to native fetal and adult-derived tissues. These analyses revealed 22 TFs whose expression did not increase in 2D differentiation systems but progressively increased in 3D culture systems and adult mature cell types. Individually overexpressing each of these TFs in immature hPSC-CMs identified five TFs (KLF15, ZBTB20, ESRRA, HOPX, and CAMTA2) as regulators of calcium handling, metabolic function, and hypertrophy. Notably, the combinatorial overexpression of KLF15, ESRRA, and HOPX improved all three maturation parameters simultaneously. Taken together, we introduce a new TF cocktail that can be used in solo or in conjunction with other strategies to improve hPSC-CM maturation and anticipate that our generalizable methodology can also be implemented to identify maturation-associated TFs for other stem cell progenies.

## INTRODUCTION

Given that heart disease represents the leading cause of death and disability worldwide,^1,2^ a renewable therapeutic source for the replacement of diseased myocardium is needed. Human pluripotent stem cell-derived cardiomyocytes (hPSC-CMs) have generated considerable interest as a regenerative therapeutic source due to their ability to be patient-specific,^3–5^ to generate an essentially limitless number of cells,^6,7^ and to be implantable within materials as a cardiac patch.^8–10^ One of the major limitations preventing translation of hPSC-CM to the clinic is the fact that these cells more closely resemble embryonic CMs than adult CMs.^11–13^ The differences between hPSC-CMs and adult CMs are numerous, including differences in morphology (e.g., sarcomere organization, cell size) and function (e.g., differences in calcium handling and electrophysiology due to the lack of t-tubules and different expression of channel genes).^14–16^ As such, numerous groups have attempted to mature hPSC-CMs via an assortment of topographical, environmental, and chemical cues.^17–22^ While these methods have improved hPSC-CM maturation, no method has successfully generated adult-like hPSC-CMs.

An alternative strategy that can be utilized separately or in conjunction with the maturation strategies described above involves the identification and overexpression of transcription factors (TFs) that drive hPSC-CM maturation.^23–27^ However, identification of maturation-associated TFs remains elusive due to two main issues. First, interrogating TF function is commonly performed via gene knockout studies,^28^ which provides evidence that a TF is involved in a developmental process but not if it is a strong regulator that drives the process. Thus, it is possible that knocking out a gene may alter a specific phenotypic response, suggesting that the gene is involved in the response, but overexpressing the same gene may not improve the phenotype. As a result, TF overexpression studies are needed to identify drivers of hPSC-CM maturation. Second, phenotypic readouts associated with maturation are often complex, preventing the use of pooled screens that are commonly utilized to rapidly screen large numbers of elements. Thus, a curated list of TFs must be tested in an arrayed format, but there is limited information as to which TFs should be screened. Indeed, the identification that HOPX overexpression improves hPSC-CM cell size, which represents one of the few known examples of a TF that drives hPSC-CM maturation, was identified by comparing RNA sequencing data of hPSC-CM maturing in 2D to adult samples.^25^ Given that maturation in 2D is limited,^29^ additional insight can be gained from analysis of hPSC-CM matured via complex strategies.

The goal of this study was to first develop a framework to systematically identify maturation-associated TFs by performing transcriptome RNAseq analysis of progressively matured hPSC-CMs across 2D and 3D differentiation systems, and further comparing these bioengineered tissues to native fetal and adult-derived tissues. Using this methodology, we were able to identify 22 TFs whose expression were not increased in 2D culture but increased in 3D and adult cells. Once we identified these TFs, we overexpressed them individually in immature hPSC-CMs and performed calcium, metabolic, and cell size analysis to identify TF drivers of multiple facets of hPSC-CM maturation. From this, we identified KLF15, ESRRA, and HOPX as key regulators of hPSC-CM calcium handling, metabolic function, and cell size. Finally, we overexpressed these TFs combinatorially to improve all three facets at once, thus developing a new TF cocktail associated with hPSC-CM maturation.

## RESULTS

### hPSC-CM differentiation in an electrically stimulated, perfused hydrogel elicits functional maturation

**FIG. 1.**
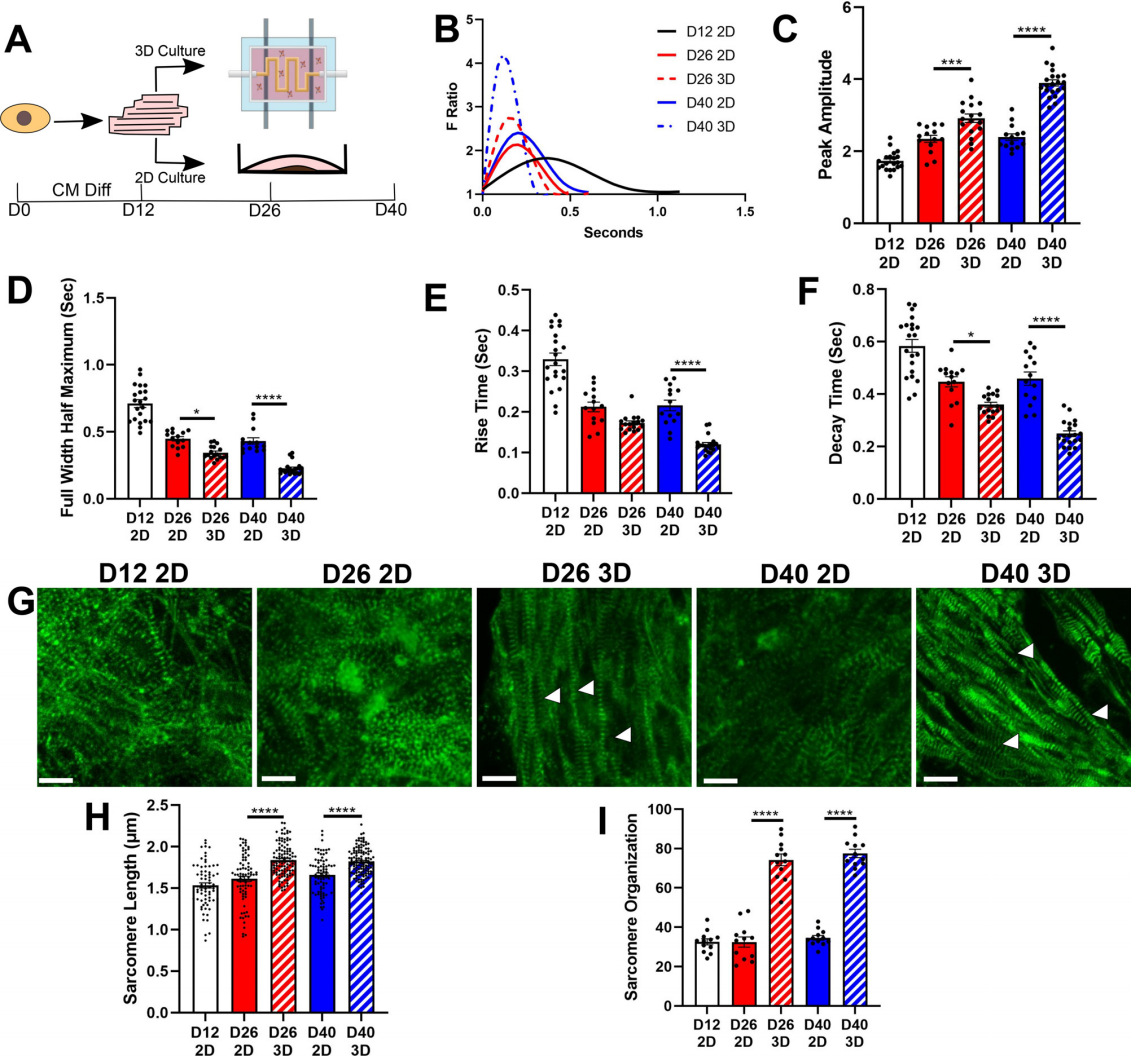
Improved phenotypic maturation of H1 hPSC-CMs in 3D hydrogel culture. (a) Schematic describing experimental design. hPSCs were differentiated into cardiomyocytes and on the 12th day of the differentiation, hPSC-CMs were either reseeded in hydrogels or left in 2D culture. Assays were performed on day 12, day 26, and day 40 to assess phenotypic maturation. (b) Representative calcium waveforms of hPSC-CMs at different time points and culture conditions. (c) Peak amplitude, (d) full width half maximum, (e) rise time, and (f) decay time of hPSC-CMs were analyzed from calcium waveforms (n = 3 differentiations, 14–21 videos). (g) Representative sarcomere staining for hPSC-CMs (scale bar = 10 *μ*m). Arrows indicate aligned sarcomeres for 3D D26 and D40 hPSC-CMs. (h) Sarcomere length (n = 3 differentiations, 70–120 sarcomeres) and (i) organization (n = 3 differentiations, 12 pictures) were analyzed from sarcomere images. ^*^p < 0.05, ^**^p < 0.01, ^***^p < 0.001, ^***^p < 0.0001, one-way ANOVA post hoc Tukey.

To identify TFs involved in hPSC-CM maturation, we first sought to develop a matured tissue engineered hPSC-CM model with improved phenotypic and transcriptomic behavior compared to 2D culture. To that end, we developed a biomimetic fibrin:gelatin hydrogel that mimicked cardiac muscle stiffness^5,30^ to mature hPSC-CMs [supplementary material Figs. 1(a) and 1(b)]. This hydrogel was seeded in a 3D printed chip that allowed for electrical stimulation between graphene rods and perfusion via the use of polyvinyl alcohol threads as a sacrificial mold [supplementary material Figs. 1(c) and 1(d)].^31^ H1 hPSCs were differentiated into hPSC-CMs using an established Wnt activator/inhibitor protocol,^32^ and on day 12 of the differentiation, hPSC-CMs were either left in 2D culture or encapsulated in hydrogels. hPSC-CMs were cultured for an additional four weeks, where hPSC-CMs encapsulated in hydrogels were subjected to a previously developed electrical stimulation program found to mature hPSC-CMs.^33^ To assess progressive maturation, hPSC-CMs were assayed for calcium handling and sarcomere length and alignment on days 12, 26, and 40 of the differentiation [Fig. 1(a)]. We observed significantly improved calcium peak amplitude and significantly reduced rise time, decay time, and full width half maximum, metrics associated with calcium handling maturation,^17–22^ over time whether cells were cultured in 2D or 3D. Importantly, we saw significantly greater increase in peak amplitude (1.63× higher for 3D D40 compared to 2D D40) and reduction in full width half maximum (0.41× lower for 3D D40 compared to 2D D40), rise time (0.55× lower for 3D D40 compared to 2D D40), and decay time (0.54× lower for 3D D40×compared to 2D D40) when hPSC-CMs were cultured in 3D compared to 2D, indicating that 3D culture better improved functional behavior of hPSC-CM [Figs. 1(b)–1(f)]. In addition, we also assessed sarcomere organization and length as organized sarcomeres are a hallmark of matured cardiomyocytes and healthy cardiac tissue, with sarcomere disarray common in diseases such as hypertrophic cardiomyopathy or fetal cardiomyocytes.^34–36^ Furthermore, sarcomere alignment is essential for the organization of other intracellular structures.^34,36^ We observed significant increases in sarcomere organization and sarcomere lengths in 3D hydrogels [Figs. 1(g)–1(i)], consistent with observations in other matured hPSC-CMs systems.^17–22^ To ensure that results we observed were consistent across different genomic backgrounds and stem cell derivation techniques, we also generated H9 and PGP1 hPSC-CMs and cultured them in 2D and 3D. We confirmed significantly increased calcium peak amplitude and significantly decreased full width half maximum, rise time, and decay time of calcium waveforms for H9 and PGP1 hPSC-CMs cultured in 3D hydrogels compared to 2D culture [supplementary material Figs. 2(a)–2(i)], thereby further validating that our 3D culture system universally improves hPSC-CM maturity beyond 2D culture.

### 3D culture promotes transcriptomic maturation and reveals maturation-associated TFs

**FIG. 2.**
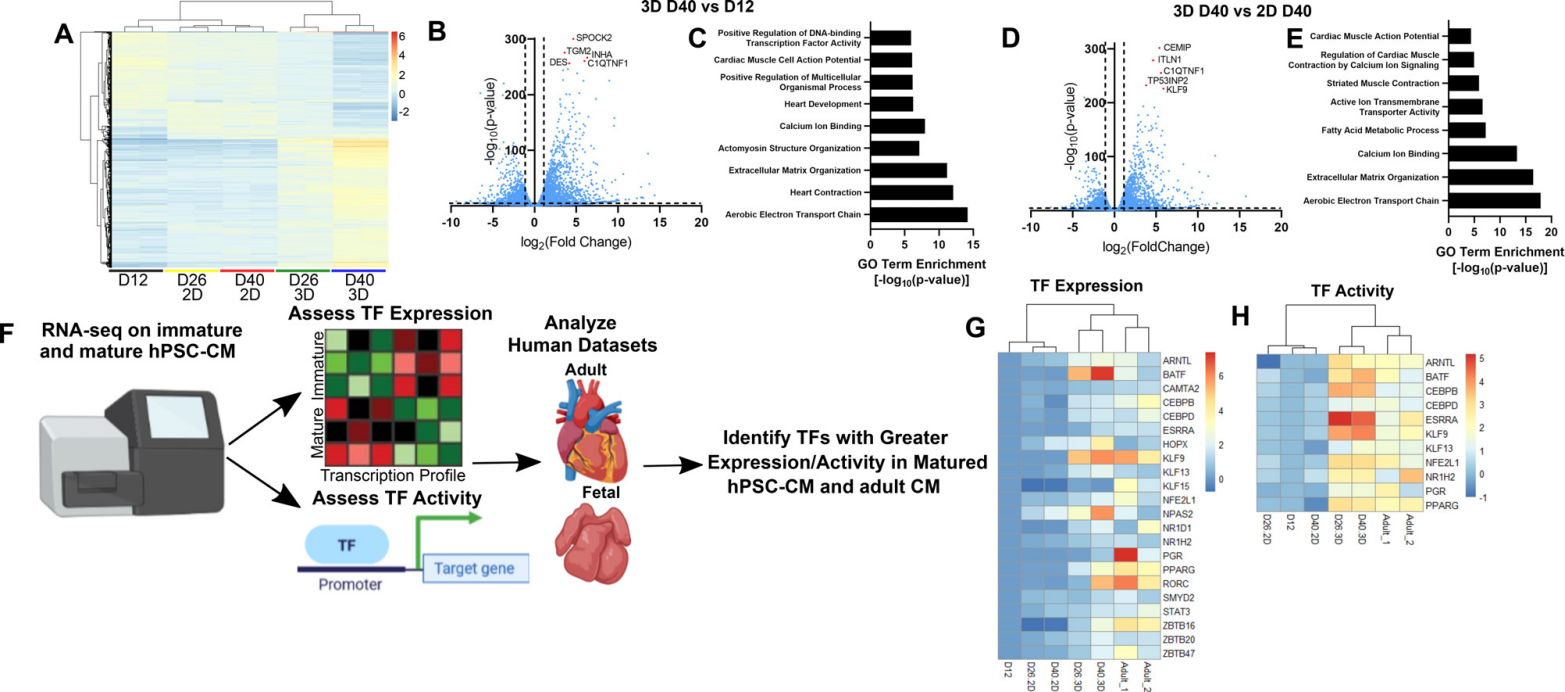
Identification of maturation-associated transcription factors expressed in hPSC-CMs in 3D hydrogel culture and adult cardiomyocytes. (a) Heatmap of the top 1000 genes with highest variance across the groups. (b) Volcano plot identifying differentially expressed genes between 3D D40 and D12 hPSC-CMs and (c) gene ontology terms associated with cardiomyocyte maturation. (d) Volcano plot identifying differentially expressed genes between 3D D40 and 2D D40 hPSC-CMs and (e) gene ontology terms associated with cardiomyocyte maturation. (f) Schematic describing RNA sequencing analysis to identify transcription factors with increased expression and activity in matured hPSC-CMs and adult CMs. (g) Log_2_ expression fold change and (h) activity of transcription factors of interest were plotted for 2D D26, 2D D40, 3D D26, and 3D D40 compared to D12 hPSC-CMs and adult compared to fetal cardiomyocytes. Fetal groups were not plotted for figure clarity.

To assess the transcriptomic changes occurring during hPSC-CM maturation in the hydrogels and identify maturation-associated TFs, we next performed RNA sequencing on D12, D26 2D and 3D, and D40 2D and 3D hPSC-CMs. D12, D26 2D, and D40 2D samples clustered more closely together compared to D26 3D and D40 3D samples, suggesting that these samples were transcriptomically distinct [Fig. 2(a)]. Notably, gene ontology (GO) analysis of differentially expressed genes (DEGs) between D40 3D samples compared to D12 and D40 2D samples identified terms associated with improved cardiac contraction, calcium handling, and metabolism [Figs. 2(b)–2(e)].

Given that 3D culture induced a distinct transcriptome, we next wanted to assess whether these changes more closely resembled proper development. We utilized two available datasets of fetal and adult CMs to compare them to 2D and 3D cultured hPSC-CMs: (1) adult left ventricle^37^ vs 9-week-old heart tissue^38^ and (2) adult vs fetal left ventricle^39^ where CMs were isolated. D40 3D samples clustered the closest to adult CMs by principal component analysis (PCA), suggesting that increased culture time and 3D culture improved hPSC-CM maturation [supplementary material Fig. 3(a)]. Next, we identified DEGs for each hPSC-CM condition compared to D12 and quantified their overlap with DEGs comparing adult CMs to fetal CMs. We observed the most overlapping DEGs with D40 3D, confirming that 3D culture indeed better resembles proper development than 2D culture [supplementary material Fig. 3(b)]. Interestingly, whereas for 3D cultures the number of overlapping genes increased over time, there were minimal improvements in overlapping genes for 2D cultures. This behavior was consistent with functional behavior, where there was limited improvement in calcium handling metrics (e.g., increased calcium peak amplitude and decreased full width half maximum, rise time, and decay time) for 2D D40 compared to 2D D26 hPSC-CMs [Figs. 1(b)–1(f), supplementary material Fig. 2]. These phenomena are also consistent with the literature, which suggests that hPSC-CMs fail to appreciably mature transcriptomically in 2D culture after 30 days of differentiation.^29,40^ To ensure that DEGs between D40 3D hPSC-CMs and D40 2D hPSC-CMs are maturation-associated genes, we assessed whether genes upregulated or downregulated in D40 3D cells compared to D40 2D cells are similarly regulated in adult CMs compared to fetal CMs. We found that DEGs for D40 3D more closely resembled DEGs between adult and fetal CMs, suggesting that 3D culture more closely mirrors fetal to adult maturation than 2D culture [supplementary material Fig. 3(c)]. In addition to the highest number of overlapping genes, we also observed upregulation of key adult-associated conduction, calcium handling, structural, and metabolic genes and downregulation of proliferation genes in 3D D40 conditions compared to the others [supplementary material Fig. 3(d)]. In the context of mitochondrial maturation, genes such as PDK4, ACSL1, CPT1A, CD36, and PPARGC1A are key to modulating lipid metabolism and shifting cardiomyocytes toward fatty acids as the main energy source, a hallmark of mature CMs.^16,41^ PPARD also induces a metabolic shift from glycolysis to fatty acid oxidation, and overexpression has been shown to mature stem cell-derived CMs.^42^ In the context of ion channels, sodium channels, such as SCN5A and SCN1B, are responsible for the upstroke velocity of the action potential. Potassium channels, such as KCND3, KCNH2, KCNJ12, induces a reduced resting membrane potential. These changes are necessary to better mirror the action potential observed in matured CMs, which are dramatically different from immature cells.^15,43^ HCN4 is responsible for the automaticity observed in hPSC-CMs. Adult ventricular cardiomyocytes do not spontaneously contract as they do not express HCN4; thus, the reduced expression is a sign of maturation. In the context of calcium handling genes, ATP2A2, CACNA1C, RYR2, PLN, CASQ2, and TRDN are key to increasing calcium stores in the sarcoplasmic reticulum as well as calcium cycling and dynamics. These genes are also involved in the formation of t-tubules, which are crucial for the effective propagation of signal in cardiac excitation–contraction coupling and are missing in immature hPSC-CMs.^44–46^ In the context of structural genes, the postnatal isoform shift from TNNI1 to TNNI3 is well characterized and associated with the organization of sarcomeres and increased contractile force. Increased expression of MYL2 compared to MYL7 is observed in matured ventricular cardiomyocytes. AKAP6, MYLK3, JPH2, and CMYA5 are all involved in sarcomeric organization. GJA5 is involved in cell–cell communication. Finally, in the context of proliferation genes, CCNB1, CDK1, AURKB, E2F1, and E2F3 are responsible for cell cycling, while CDKN1A and CDKN2A are cell cycle inhibitors that prevent proliferation.^47,48^ Within these genes, we observed differential timing of upregulation, where some have similar expression levels between day 26 and day 40, some increase in expression over time, and some are only upregulated at day 40. Together, these analyses helped identify maturation-associated genes and their temporal pattern of expression.

Next, we wanted to identify TFs with high expression and activity in matured hPSC-CMs and adult CMs. Given the difficulty in procuring human cardiac tissue at various developmental stages, we mainly focused on the early stage changes in TF expression and activity that we observed in matured hPSC-CMs compared to immature cells. Importantly, since these TFs are also upregulated in adult CMs compared to fetal CMs, they most likely play some role in establishing or maintaining the adult CM transcriptome. These TFs were identified using DESeq2 for assaying expression levels^49^ and DoRothEA,^50^ which approximates TF activity based on expression levels of genes known to be activated by TF, for assaying activity levels [Fig. 2(f)]. These interactions are determined by literature, inferences from gene expression, Chip-Seq Peaks, and TF binding motifs on promoters. From this, we curated a list of 12 genes with high expression and activity in our data and adult tissue. Given that DoRothEA has a limited set of TF-gene interactions, we also included genes that did not have an activity score but did have high transcript expression and either came from the same families as the 12 selected genes (e.g., KLF family) or were genes with known interactions with important CM genes, together giving us a total of 21 TFs. In addition, given that members of the KLF family were enriched in our analysis, we were interested in casting a wider net for other members of interest. Although KLF15 was not improved in our system, we included it in the screen as it was significantly upregulated in adult CMs vs fetal CMs for both datasets we analyzed, and mouse studies suggested that KLF15 has some role in maturation based on a knockout study.^51^ We reasoned that since our matured hPSC-CMs are still immature compared to adult CMs, it makes sense to include additional TFs expressed in adult CMs but not matured hPSC-CMs. We confirmed expression and activity of these TFs in hPSC-CMs cultured in 3D were more closely clustered with adult CMs compared to those cultured in 2D [Figs. 2(g) and 2(h)]. In addition, we confirmed increased mRNA expression of these TFs in D40 3D PGP1 and H9 hPSC-CMs compared to D40 2D and D12, highlighting that these TFs are universally upregulated in our maturation system [supplementary material Fig. 4(a)]. Finally, to ensure these TFs are upregulated in previously published maturation models, we next performed TF expression and activity analysis on six different maturation model sets.^17,18,20,22,52,53^ Ronaldson-Bouchard *et al.* and Zhao *et al.* utilized electrical stimulation to induce maturation. Ichimura *et al.* implanted hPSC-CMs into rat hearts to utilize the *in vivo* environment to mature cells. Branco *et al.* and Giacomelli *et al.* utilized 3D culture to mature hPSC-CMs. Giacomelli additionally co-cultured CMs with cardiac fibroblasts and endothelial cells. Finally, Feyen *et al.* matured hPSC-CMs using chemical factors. Thus, these models were chosen as they utilized several different methods to mature hPSC-CMs, which will give the largest breadth of potential maturation-associated TFs. We observed upregulation of expression and/or activity for all genes in at least one model, suggesting that these TFs may be drivers of hPSC-CM maturation [supplementary material Fig. 4(b)]. Taken together, in this curated list of TFs, while some have known effects on heart development, largely via animal or cell knockout studies, the effect of several others on heart development is unknown (supplementary material Table 2).

### KLF15, ESRRA, and HOPX overexpression improve hPSC-CM calcium handling, mitochondrial function, and cell size

**FIG. 3.**
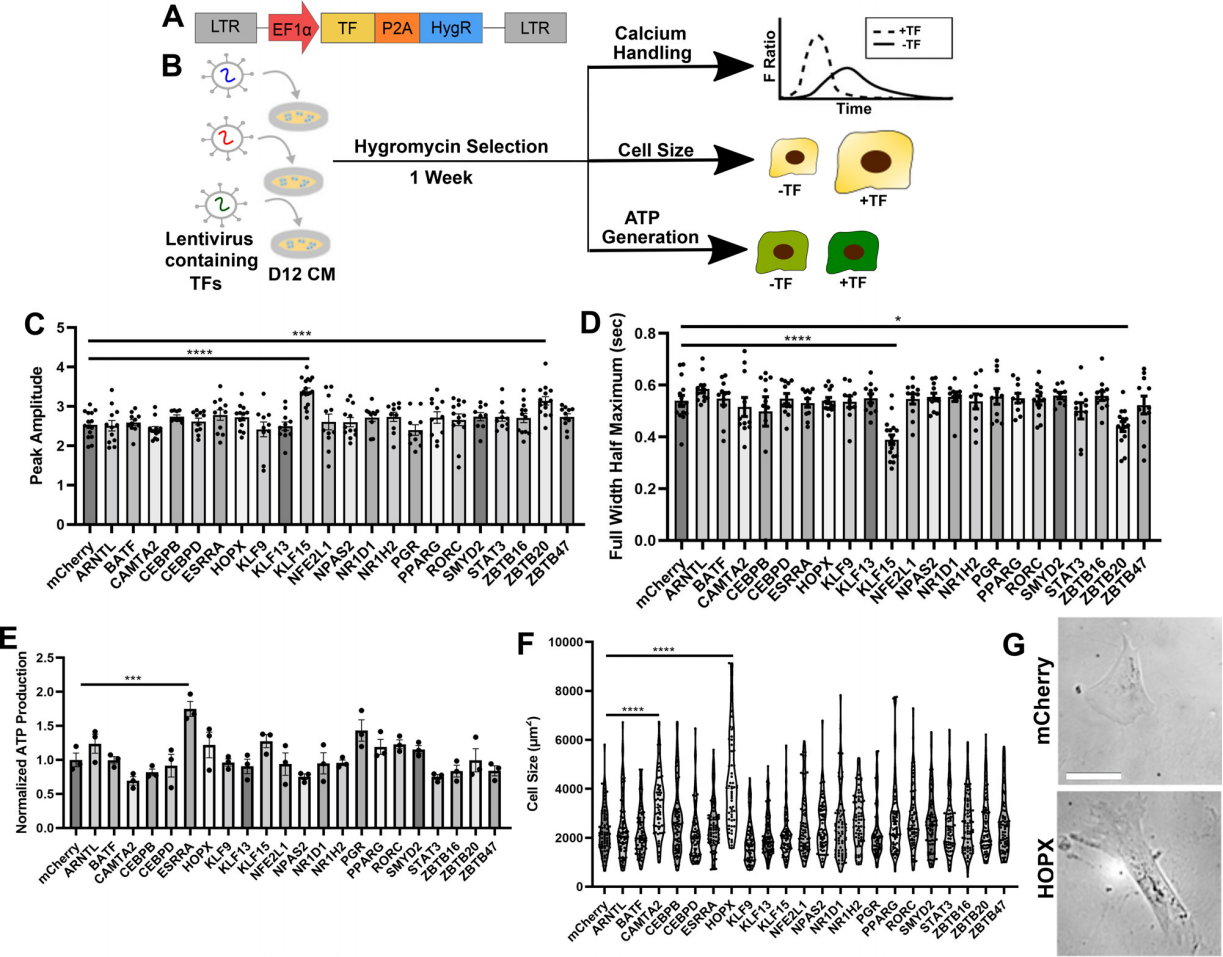
Identification of KLF15, ESRRA, and HOPX as key regulators of hPSC-CM calcium handling, metabolic function, and cell size. (a) Schematic of the lentiviral vector to induce transcription factor overexpression and hygromycin resistance. (b) Experimental setup for the arrayed screen. On day 12 of the differentiation, hPSC-CMs were re-plated and transduced with lentivirus corresponding to one transcription factor. Hygromycin selection started after two days, and cells were assayed for calcium handling, ATP generation, and cell size one week later. (c) Peak amplitude and (d) full width half maximum of hPSC-CMs overexpressing mCherry or TFs were analyzed from calcium waveforms (n = 3 differentiations, 9–16 videos). (e) ATP production normalized to cell number was plotted for hPSC-CMs (n = 3 differentiations). (f) Cell size was plotted for hPSC-CMs (n = 3 differentiations, 57–81 cells) with representative images of mCherry and HOPX-overexpressing hPSC-CMs, shown in (g) (scale bar = 100 *μ*m). ^*^p < 0.05, ^**^p < 0.01, ^***^p < 0.001, ^***^p < 0.0001, one-way ANOVA post-hoc Dunnett's test.

To identify direct modulators of hPSC-CM maturation, we next cloned the TF sequences from the curated list above into lentiviral constructs containing a hygromycin resistance gene [Fig. 3(a)]. After generating the lentivirus, we performed an arrayed screen where day 12 H1 hPSC-CMs were transduced with individual lentiviruses. After two days, hygromycin treatment was started, and after one week, cells were assayed for calcium handling, cell size, and ATP generation [Fig. 3(b)]. Compared to respective mCherry controls, we observed that hPSC-CMs overexpressing KLF15 exhibited significantly improved calcium flux amplitude and reduced full width half maximum [Figs. 3(c) and 3(d)], hPSC-CMs overexpressing ESRRA showed increased ATP generation [Fig. 3(e)], a surrogate for mitochondrial function^54^), and hPSC-CMs overexpressing HOPX were significantly larger [Figs. 3(f) and 3(g)]. In addition, we observed significantly reduced rise and decay times for calcium waveforms for KLF15-overexpressing cells compared to control [supplementary material Figs. 5(a) and 5(b)], and HOPX-overexpressing cells exhibited significantly reduced circularity and increased aspect ratio compared to mCherry overexpressing cells, which is consistent with mature CMs that are rectangular in shape [supplementary material Figs. 5(c) and 5(d)].^55–57^ In addition, we observed modest improvements in calcium handling (e.g., increased peak amplitude, reduced full width half maximum, and reduced decay time) for ZBTB20-overexpressing hPSC-CMs and cell size for CAMTA2-overexpressing hPSC-CMs.

### Combinatorial TF overexpression improves multiple aspects of hPSC-CM maturation

**FIG. 4.**
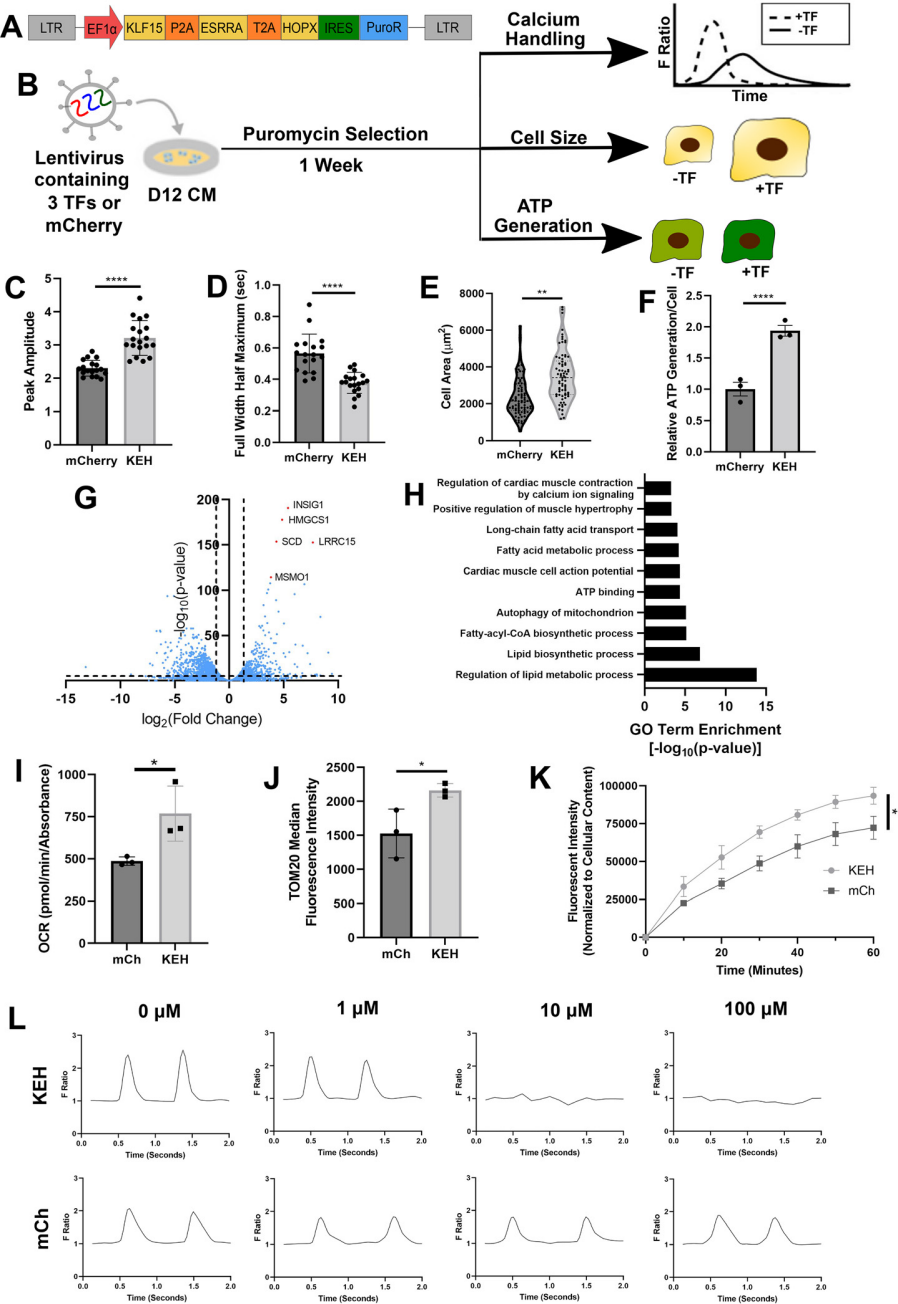
Combinatorial overexpression induces phenotypic and transcriptomic improvements in hPSC-CMs. (a) Tricistronic vector allowing for simultaneous overexpression of KLF15, ESRRA, and HOPX (KEH), along with puromycin selection marker. (b) D12 hPSC-CMs were transduced with lentiviral constructs overexpressing KEH or mCherry, followed by puromycin selection after two days, and assays after one week. (c) Peak amplitude and (d) full width half maximum of hPSC-CMs overexpressing mCherry or TFs were analyzed from calcium waveforms (n = 3 differentiations, 18–19 videos). (e) Cell size was plotted for hPSC-CMs (n = 3 differentiations, 74–78 cells). (f) ATP production normalized to cell number was plotted for hPSC-CMs (n = 3 differentiations). (g) Volcano plot identifying differentially expressed genes between KEH and mCherry-overexpressing hPSC-CMs and (h) gene ontology terms associated with cardiomyocyte maturation were plotted. (i) Maximal oxygen consumption rate (OCR), as measured by a Seahorse analyzer, and normalized to cell number was plotted for hPSC-CMs (n = 3 differentiations). (**j**) TOM20 fluorescent intensity, a metric of mitochondrial content, was measured by FACS and plotted (n = 3 differentiations). (k) Fatty acid uptake over time was normalized to cell content and plotted (n = 3 differentiations). (l) Representative calcium waveforms for cells treated with various concentrations of tetrodotoxin. KEH-overexpressing cells were unable to beat in 10 and 100 *μ*M tetrodotoxin containing media, whereas mCherry cells were unaffected (n = 3 differentiations). ^*^p < 0.05, ^**^p < 0.01, ^***^p < 0.001, and ^***^p < 0.0001, two-tailed unpaired Student's t-test.

Given that no single TF improved more than one aspect of hPSC-CM maturation, we decided to create a tricistronic vector overexpressing KLF15, HOPX, and ESRRA (KEH) followed by an IRES sequence to drive puromycin resistance to simultaneously modulate all three aspects of hPSC-CM maturity [Fig. 4(a)]. The experiment was performed as previously described, where H1 D12 hPSC-CMs were transduced with lentivirus, selected with puromycin after two days, and assays were performed one week later [Fig. 4(b)]. Notably, we observed significantly improved calcium amplitude [Fig. 4(c)], reduced full width half maximum [Fig. 4(d)], increased cell size [Fig. 4(e)], and increased ATP generation [Fig. 4(f)] in hPSC-CMs overexpressing KEH compared to mCherry controls. In addition, we observed significantly decreased rise time [supplementary material Fig. 6(a)] and decay time [supplementary material Fig. 6(b)] of calcium waveforms for KEH-overexpressing hPSC-CMs, increased aspect ratio [supplementary material Fig. 6(c)], and reduced circularity [supplementary material Fig. 6(d)]. We observed similar improvements in calcium handling, ATP generation, and morphological characteristics [supplementary material Figs. 7(a)–7(h)] in PGP1 hiPSC-CMs, suggesting that the results are not cell line-specific.

To understand how KEH overexpression induces hPSC-CM maturation, we next performed RNA sequencing on KEH and mCherry overexpressing cells. GO analyses pointed to strong improvements in lipid metabolic function as well as cell growth and cardiac conduction [Figs. 4(g) and 4(h)]. To assess whether these transcriptomic changes mirrored proper development, we compared KEH and mCherry-overexpressing CMs to fetal and adult datasets and observed that KEH-overexpressing cells clustered closer to adult CMs compared to mCherry cells [supplementary material Fig. 8(a)]. Next, we examined whether upregulated and downregulated genes in KEH-overexpressing hPSC-CMs were similarly regulated in adult CMs compared to fetal CMs. KEH upregulated genes were largely upregulated in adult CMs, while KEH downregulated genes were also mostly downregulated in adult CMs, suggesting a more mature transcriptome for KEH-overexpressing hPSC-CMs [supplementary material Fig. 8(b)]. Indeed, we observed strong upregulation of numerous adult-associated metabolic genes and downregulation of fetal-associated genes [supplementary material Fig. 8(c)]. Among these genes, PDK4, which increases the reliance of the heart on fatty acid oxidation for energy production, is a direct target of KLF15.^58^ Similarly, KLF15 cooperates with PPARA to regulate cardiomyocyte lipid gene expression.^59^ ESRRA knockdown is associated with downregulation of ACSL1 and CPT1A, which are upregulated in KEH overexpressing cells.^60^ Finally, we observed upregulation of PPARD, which has recently been shown to induce metabolic and contractile maturation in hPSC-CM.^42^ To further validate improved metabolic function in KEH-overexpressing cells, we quantified oxygen consumption rate (OCR) using a Seahorse Analyzer and observed a significant increase in maximal respiration rates in KEH-overexpressing cells compared to control cells [Fig. 4(i)]. This finding is consistent with other maturation studies and represents a shift toward oxidative phosphorylation to provide energy to cells.^17,22,61^ Given these results, we were interested in assessing whether total mitochondrial content is increased in KEH-treated cells. To assess mitochondrial content, we stained for the mitochondrial marker TOM20 and measured the amount by FACS. Significantly increased TOM20 intensity in KEH-overexpressing cells compared to control cells indicate increased mitochondrial content, which is consistent with prior studies [Fig. 4(j)].^17^ Next, we investigated fatty acid uptake capacity, a hallmark of cardiomyocyte maturation is the switch from glucose to fatty acids as an energy source. Indeed, fatty acid supplementation to media has been shown to improve stem cell-derived cardiomyocyte maturation.^61^ Furthermore, KEH-overexpressing cells exhibited increased CD36 expression, a key protein involved in fatty acid uptake.^42,61^ Indeed, H1 and PGP1 KEH-overexpressing cells exhibit a significantly higher fatty acid uptake capacity compared to control cells [Fig. 4(k), supplementary material Fig. 7(i)]. Taken together, these results demonstrate that KEH-overexpressing cardiomyocytes have a more mature metabolic state as well as the metabolic machinery needed to switch from glucose to fatty acids as an energy substrate. With regard to ion channel expression, we observed upregulation of numerous potassium, sodium, and calcium related channels [supplementary material Fig. 8(d)]. KCNIP2, which is directly controlled by KLF15, modulates the Kv4 family of potassium channels that together define the fast transient outward potassium current (I_to,f_) and maintains early cardiac repolarization.^62,63^ hPSC-CMs largely fail to express I_to,f_, thus providing a direct link between KLF15 overexpression and improved ion channel function.^64^ In addition, we observed upregulation of numerous sodium channels in KEH-overexpressing cells. One of the key switches as cardiomyocytes mature is the reliance on sodium to induce action potentials instead of calcium.^17^ Indeed, a previous group has demonstrated that addition of the sodium channel blocker tetrodotoxin prevents beating in mature cells at 10 *μ*M, whereas immature cells are resistant even at 100 *μ*M.^17^ We observed a similar trend, where H1 and PGP1 KEH-overexpressing cells failed to beat with the addition of 10 and 100 *μ*M tetrodotoxin, while control cells were largely unaffected [Fig. 4(l), supplementary material Fig. 7(j)]. With regard to hypertrophy, we observe upregulation of Ca^2+^/calmodulin-dependent protein kinase (CAMK) and NFAT pathway activity, which are known to act in parallel to induce cardiac hypertrophy [supplementary material Fig. 8(e)].^65,66^ We also observed upregulation of key structural genes, such as titin,^67^ spectinβ4,^68^ and rho-associated protein kinases,^69^ which contribute to actin cytoskeleton structure and cardiac contraction. In addition to these changes, we observed key isoform switches, such as the ratio of MYL2 to MYL7 and TNNI3 to TNNI1 gene expression, and strong downregulation of cell cycle genes and upregulation of cell cycle inhibitor genes, suggesting that the cells are becoming less proliferative [supplementary material Fig. 8(f)]. Reduced proliferation is one of the key changes that occur as cardiomyocytes mature, with adult cardiomyocytes being post-mitotic.^15,16,70^ Taken together, these data suggest that hPSC-CM overexpressing KEH exhibit functional and transcriptional maturation across multiple facets of cardiomyocyte function.

## DISCUSSION

Numerous strategies have been explored to improve hPSC-CM maturity; however, none of these strategies has individually fully matured cells.^16,61,70–74^ Most likely, a combinatorial approach of tissue and genome engineering is needed to recapitulate proper development. As such, there is a great need for scalable and broadly applicable strategies that are complementary to current strategies. Although TF overexpression represents such a strategy, identifying TFs that drive maturation has proven elusive. This is due in part to a lack of understanding of the breadth of maturation-associated TFs as well as a method to screen the effects of these TFs. In this study, we performed transcriptomic analysis on a progressively matured hPSC-CM tissue engineering model and identified 22 TFs whose expression was not increased in 2D culture but progressively increased by 3D culture and adult CMs. We next performed a TF overexpression arrayed screen on immature day 12 hPSC-CMs and assessed calcium handling, metabolism, and cell size. We focused on day 12 hPSC-CMs, as studies have indicated that early stage (e.g., day 12) hPSC-CMs may be more responsive to perturbations than late stage cells (e.g., day 28).^22^ Thus, by intervening earlier, we wanted to establish a TF cocktail that more strongly drives hPSC-CMs toward a more mature phenotype. We identified KLF15, ZBTB20, ESRRA, HOPX, and CAMTA2 as drivers of hPSC-CM calcium handling, metabolism, and hypertrophy maturation. The ability to identify five drivers of hPSC-CM maturity represents the strength of this approach, and although KLF15 was identified using an unsupervised approach, its inclusion was based on our computational analysis that identified similar family members, such as KLF9 and KLF13. Although ZBTB20 and CAMTA2 represent novel hits in the context of hPSC-CM maturation, we focused on combining KLF15, ESRRA, and HOPX (KEH) due to size constraints of the lentivirus. By combining KEH in a tricistronic vector, we were able to simultaneously improve functional and transcriptomic behaviors for all three aspects. Whereas previous studies have largely focused on a single aspect of hPSC-CM maturity, e.g., cell size or metabolism,^25,42^ we focused on improving multiple aspects of maturity simultaneously by combining multiple TFs. Numerous papers have demonstrated that different perturbations induce different facets of maturation, suggesting the need to properly activate multiple signaling networks to improve maturation.^15,16,75^

Transcriptomic analysis of KEH-overexpressing cells provides mechanistic insight into how it induces maturation. This study is the first to identify KLF15 as a modulator of hPSC-CM calcium handling. Although KLF15 has largely been investigated in the context of its metabolic effects,^58,76^ little is known about the effects of KLF15 on calcium handling. However, the direct interaction of KLF15 with KCNIP2 provides a potential link that ties KLF15 to improved ion channel expression in hPSC-CMs.^63,76,77^ As mentioned, KCNIP2 regulates I_to,f_, which has several interrelated roles in cardiomyocyte function; it contributes to the action potential waveform and rate-dependent action potential properties as well as to excitation–contraction coupling by influencing calcium influx.^78–80^ However, it appears that KCNIP2 may not be limited to just modulating the Kv4 family. KCNIP2 knockdown reduces expression of SCN5A and KCND3, which are both upregulated in KEH-overexpressing cells. Surprisingly, KCNIP2 knockdown also leads to reduced calcium transient amplitude and prolonged transient duration via reduction of ryanodine receptor activity.^79,80^ Given that hPSC-CMs fail to exhibit I_to,f_, largely due to much lower expression of KCNIP2 than adult CM,^64,81,82^ it is possible that KLF15-mediated overexpression of KCNIP2 is driving the improved calcium handling we observed. With regard to metabolic behavior, we observed improved ATP generation and strong upregulation of genes associated with lipid metabolic function with KEH than with either ESRRA or KLF15 alone. Both of these genes are associated with improved lipid metabolism,^58–60^ suggesting a synergistic effect.

Taken together, we have developed a pipeline for systematically identifying maturation-associated TFs, which can also be easily applied to other stem cell-derived progenies. By curating a list of TFs, we were able to conduct an arrayed screen with complex calcium handling, metabolic, and hypertrophy readouts. From this, we were able to identify a cocktail of TFs that improve hPSC-CM maturation. Looking forward, this cocktail can be used in solo or combined with additional TFs or strategies to further improve hPSC-CM maturation.

## METHODS

### Cardiomyocyte differentiation

H1, H9, and PGP1 hPSCs were cultured on growth factor-reduced Matrigel (Corning, 354230)-coated well plates with mTeSR1 media (STEMCELL Technologies, 85850). For CM differentiations, hPSCs were passaged with Accutase (Innovative Cell Technologies, AT104) and treated with small molecular inhibitors as previously described.^32^ Briefly, hPSCs were grown in mTeSR1 media (STEMCELL Technologies, 85850) until 90% confluence. Cells were then treated with 12 *μ*M CHIR99021 (Tocris Bioscience, 4423) in RPMI 1640 media (Gibco, 11875) containing B27 supplement (Thermo, 17504), defined as day 0 of the differentiation. On day 1, CHIR99021 was removed and cells were cultured in RPMI 1640 media containing B27 minus insulin supplement (Thermo, A18956). On day 3, combined media consisting of 1:1 ratio of used media and fresh RPMI 1640 containing B27 minus insulin media and 5 *μ*M IWP2 (Tocris Bioscience, 3533) were added to cells. Media were changed to RPMI 1640 containing B27 minus insulin on day 5, followed by media changes with RPMI media containing B27 supplement on days 7, 9, and 11. Cardiomyocytes were used for experiments as described on day 12.

### 3D printing and perfusion setup

Long-term perfusion of tissue constructs was achieved via a three-component setup containing a media reservoir, a 3D printed flow chamber, and a peristaltic pump (Watson Marlow, 205U) connected via silicon tubing (McMaster Carr). Flow chambers were constructed via extrusion-printing of silicon (Dow Corning, Sylgard SE1700) onto glass slides. CAD drawings were created in CADFusion to allow for an inlet and outlet port, gaps for the insertion of graphene rods, and posts that keep the fibrin hydrogel contained between the rods.

### Perfused hydrogel formation

Stock solutions of gelatin, calcium chloride, thrombin, bovine plasma fibrinogen, and transglutaminase were prepared as previously described.^31^ To allow for perfusion through the hydrogel, poly(vinyl) alcohol threads (Solvron, Nitivy Co. 62T Type S) were wrapped around the 3D printed posts as a sacrificial mold. Hydrogels were created by first resuspending hPSC-CMs at 10 million cells/mL in fibrinogen (20 mg/ml), transglutaminase (2 mg/ml), calcium chloride (2.5 mM), gelatin (1.5 wt. %/ml), and RPMI1640 containing B27 supplement and incubating for 30 minutes at 37 °C. Next, Matrigel (5 mg/ml) and thrombin (2 U/ml) are added and the mixture is pipetted over the Solvron threads within the 3D printed posts. After complete gelation, the medium is added.

### Atomic force microscopy

Hydrogel stiffness measurements were determined by atomic force microscopy (MFP-3D Bio, Asylum Research) with a silicon nitride cantilever (NanoAndMore USA Corporation, cat # PNP-TR). Tip deflections were converted to indentation force for all samples using their respective tip spring constants and Hooke's Law. All AFM data were analyzed using custom-written code in Igor Pro to determine Young's Modulus as previously described based on a Hertz model.^5,83^ Hydrogel formulation tested included 10 mg/ml fibrinogen hydrogel, a 20 mg/ml fibrinogen hydrogel, and a hydrogel with no fibrinogen and 7.5 wt. %/ml gelatin. All other components were kept at the same concentration.

### hPSC-CM maturation culture

On day 12 of the cardiomyocyte differentiation, cells were lifted from plates using Accutase and encapsulated in hydrogels as described above. Flow was started the next day, while electrical stimulation was started after 7 days. To perform electrical stimulation, Arduino Uno microcontrollers equipped with motor shields were connected to a 12 V power supply. The microcontrollers were programmed to provide a stimulation program previously shown to induce cardiomyocyte maturation.^22^ Stimulation was started at a frequency of 2 Hz and increased to 6 × 0.33 Hz per day, followed by one week at 2 Hz. Arduino Uno microcontrollers were connected to graphene rods using electrical wire.

### Immunofluorescence and sarcomere analysis

Cells and hydrogels were fixed in 4% paraformaldehyde in PBS for 15 and 60 min, respectively. Samples were then permeabilized using 0.25% w/v Triton X-100 for 15 min at room temperature. Samples were then incubated in blocking buffer (1% BSA, 2.5% goat serum, and 0.5% Tween) for two hours at room temperature. Samples were incubated overnight at 4C with anti-α-actinin antibody (1:100, Thermo EA-53) or TOM20 antibody (1:100, Cell Signaling D8T4N) followed by Alexa Fluor 488 conjugated goat anti-mouse secondary antibody (1:1000, Thermo, cat # A-11001) or Alexa Fluor 488 conjugated goat anti-rabbit secondary antibody (1:1000, Thermo, cat #11008) for one hour at room temperature. Finally, samples were incubated in 4′,6-Diamidino-2-Phenylindole (DAPI, 1:5000, Thermo, cat # D1306) in H_2_O for 10 minutes at room temperature. Alpha-actinin images were taken using a 63× objective on a Nikon Eclipse TI fluorescence microscope. TOM20 intensity was measured using a BD LSRFortessa Cell Analyzer.

Sarcomere length was quantified by measuring the distance between sarcomeres using Fiji (NIH). Sarcomere alignment was assessed using a scanning gradient Fourier transform method that incorporates gradient analysis along with fast Fourier transforms to determine regions of sarcomere organization within individual and a population of cells. This provides an overall direction of pattern for the sarcomeres. Then the percentage of sarcomeres aligned within 20% of this principal axis was calculated as the sarcomere alignment.^84^

### Calcium handling

Calcium imaging was performed by adding media containing 1 *μ*M Fluo-4 AM (Thermo Fisher) for 20 minutes to cells. The media were changed, and cells were incubated for an additional 30 minutes to allow for complete de-esterification of intracellular AM esters. For tetrodotoxin assays, 0, 1, 10, and 100 *μ*M was added at this point. Videos were captured using a 20× objective on a Nikon Eclipse TI fluorescent microscope. Videos were imported into Fiji (NIH) and five cells from each video were randomly selected, and the mean fluorescent intensities were recorded. Ca^2+^ tracings were then analyzed using custom-written code in MATLAB to determine mean peak amplitude.^5^ Full width half maximum, rise time, and decay time were calculated using Clampfit 10.7 software (Molecular Devices).

### Morphological characterization

Brightfield imaging of H1 hPSC-CMs were taken at 20× magnification and analyzed using Fiji (NIH). The edges of cells were traced, and area, aspect ratio, and circularity were quantified.

### ATP generation

hPSC-CMs were seeded in 96 wells in two sets of triplicate wells. One set was used to determine ATP generation, and one set was used to normalize to DNA content using crystal violet staining. ATP generation was performed using CellTiter-Glo 2.0 (Promega), and luminescence was measured on a SpectraMax iD5 Multi-Mode Microplate Reader (Molecular Devices) per manufacturer's instructions. DNA content was assessed by staining cells with crystal violet, solubilizing using 1% sodium dodecyl sulfate, and absorbance was measured at 595 nm on the plate reader. Luminescence values were divided by the absorbance values, and all values were normalized to mCherry values.

### Seahorse Mito stress test

H1 hPSC-CMs were plated on Matrigel-coated Seahorse XFp cell culture microplates at 20,000 cells per well. Cells were treated with lentivirus and cultured as described. One hour prior to the assay, media were switched to XF DMEM media containing 1 mM pyruvate, 2 mM glutamine, and 10 mM glucose. Oxygen consumption rate (OCR) was measured sequentially after addition of 1.0 μM oligomycin, 0.5 *μ*M FCCP, and 0.5 *μ*M rotenone. OCR was normalized to cellular content using crystal violet assay.

### Fatty acid uptake

hPSC-CMs were plated on Matrigel-coated 96 well plates, treated with lentivirus, and cultured as described. Fatty acid uptake was assessed using the QBT Fatty Acid Uptake Kit per manufacturer's instructions. Values were taken at 10-minute increments and normalized to cellular content using crystal violet assay.

### Generation of TF overexpressing plasmids

The lentiviral TF-Hygro vector contained the EF1a promoter, mCherry transgene flanked by BamHI restriction sites, followed by a P2A peptide, and hygromycin resistance enzyme gene (Addgene #120426). Each TF in the library was individually inserted in place of the mCherry transgene. TF sequences were PCR amplified from the DNASU 90/90 Human ORFeome V1-Transcription Factor Subcollection and inserted into the TF-Hygro vector via Gibson assembly. Combinatorial TF-Puro vector was constructed in a similar manner but with the starting vector containing an IRES sequence followed by puromycin resistance gene (Addgene #26777).

### Lentivirus production

HEK293T cells were maintained in DMEM supplemented with 10% FBS. Cells were seeded into a 15 cm dish such that cells were 60%–70% confluent. 36 *μ*l Lipofectamine 2000 (Life Technologies) was added to 1.5 ml of Opti-Mem (Life Technologies) in one tube and 3 *μ*g of pMD2.G (Addgene #12259), 12 *μ*g of pCMV delta R8.2 (Addgene #12263), and 9 *μ*g of each individual vector was added to 1.5 ml of Opti-Mem in another tube. After 5 min of incubation, tubes were mixed and incubated for 30 min. The solution was then added dropwise to the 15 cm dish. Viral-containing supernatant was harvested after 48 and 72 h, filtered with Steriflip (Millipore), concentrated to 1 ml using Amicon Ultra-15 centrifugal ultrafilters with a 100 000 NMWL cutoff (Millipore), and frozen at −80 °C.

### Lentivirus transduction

On day 12 of the differentiation, H1 hPSC-CMs were reseeded in 24 well plates. The next day, cells were treated with 100 *μ*l lentivirus and 8 *μ*g/ml polybrene (Millipore). After two days, 50 *μ*g/ml hygromycin or 1 *μ*g/ml puromycin containing the media was added to cells. After one week of treatment, cells were assayed for calcium handling, cell size, or ATP generation.

### RNA sequencing analysis

RNA was isolated using the RNeasy Mini Kit (Qiagen) per manufacturer's instructions. RNA libraries were generated from 300 ng of RNA using the NEBNext Ultra II Directional RNA Library Prep Kit for Illumina (New England Biolabs) and sequenced on an Illumina NovaSeq platform.

Fastq files were mapped to GRCh38 and read counts quantified using Gencode v28 and STAR aligner. Read counts were normalized using DESeq2. Hierarchical clustering between replicates was performed based on the average distance in relative expression levels of all expressed genes across replicates. Principal component analysis was performed using relative expression levels of all genes across replicate. Relative expression profiles and differentially expressed gene lists were subsequently generated using the DESeq2 pipeline. Differentially expressed gene cutoff was calculated with a log_2_(fold change) expression of greater than 0.5 or less than -0.5 and a p-value cutoff of 0.005. Gene ontology analysis was performed using Enrichr.^85^ TF activity was analyzed using DoRothEA. We only assessed activity for TFs with confidence scores of A, B, and C.

To identify TF targets, we used DESeq2 analysis to identify all TFs with at least 1.5-fold overexpression for D40 3D cells compared to D40 2D and D12 cells. DoRothEA analysis was used to identify all TFs with at least 1.5-fold greater activity in D40 3D cells compared to D40 2D cells. We next identified similar TFs in adult vs fetal cardiomyocytes using the same criteria. We compared non-failing adult left ventricles (PRJNA477855) to 9-week-old hearts (PRJEB27811), and adult left ventricles were compared to fetal left ventricles where cardiomyocytes were sorted (GSE156702). From here, we curated a list of 12 TFs with higher expression and activity in both D40 3D cells and adult cardiomyocytes compared to their respective groups. We next included an additional nine TFs with high expression, but not activity score based on having similar behavior to the 12 genes selected or genes with suggested effects in the heart. Finally, we included KLF15 as multiple KLF genes showed up in our analysis, and it was strongly upregulated in adult cardiomyocytes. We then assessed whether the chosen TFs had higher expression or activity in other hPSC-CM maturation models. We chose six models and counted how many times a TF met the criteria listed above.

### Quantitative PCR

RNA was extracted using the RNeasy Mini Kit (Qiagen) per manufacturer's instructions, and cDNA was prepared using the Protoscript II First Strand cDNA synthesis kit (New England Biolabs). qRT-PCR reactions were then set up containing 2 *μ*l cDNA, 400 nM of each primer, 2× iTaq Universal SYBR Supermix (Bio-Rad), and H_2_O up to 20 *μ*l. qRT-PCR was performed (95 °C for 3 min; 95 °C for 5 s, followed by 60 °C for 30 s, for 40 cycles) using a CFX Connect Real Time PCR Detection System (Bio-Rad). The results were normalized to the housekeeping gene DDB1. Relative mRNA expression was determined by the comparative cycle threshold (ΔΔCT) method. Primers used in the study are listed in supplementary material Table 1.

### Statistical analysis

All statistical analyses were performed using GraphPad Prism. Data are represented as mean ± standard error of the mean.

## SUPPLEMENTARY MATERIAL

See the supplementary material for additional figures (supplementary material Figs. 1–6), supplementary material Table 1 (primers used for qPCR), and supplementary material Table 2 (contains the known interactions that each TF has in cardiomyocytes).

## Data Availability

The data that support the findings of this study are available from the corresponding authors upon reasonable request.
